# Morbidity and mortality in premature or low birth weight patients with congenital heart disease in three European pediatric heart centers between 2016 and 2020

**DOI:** 10.3389/fped.2024.1323430

**Published:** 2024-04-11

**Authors:** Alexandra De Silvestro, Bettina Reich, Sarah Bless, Julika Sieker, Willemijn Hollander, Karen de Bijl-Marcus, Cornelia Hagmann, Joppe Nijman, Walter Knirsch, the European Association Brain in Congenital Heart Disease, Mirielle N. Bekker

**Affiliations:** ^1^ Pediatric Cardiology, Pediatric Heart Center, University Children’s Hospital, University of Zurich, Zurich, Switzerland; ^2^ Children’s Research Center, University Children’s Hospital Zurich, Zurich, Switzerland; ^3^ Pediatric Cardiology and Congenital Heart Disease, German Heart Center, Munich, Germany; ^4^ Department of Pediatric Intensive Care, University Medical Center Utrecht, Utrecht, Netherlands; ^5^ Department of Neonatology, Wilhelmina Children’s Hospital, Utrecht University, Utrecht, Netherlands; ^6^ Department of Neonatology and Pediatric Intensive Care, University Children’s Hospital, University of Zurich, Zurich, Switzerland

**Keywords:** preterm, low birth weight, congenital heart disease, mortality, morbidity, cardiac intervention, surgery, complication

## Abstract

**Background:**

The treatment of preterm and low birth weight (LBW) neonates born with congenital heart disease (CHD) requiring early cardiac intervention remains challenging. We aimed to analyze morbidity and mortality in this combined high-risk patient group.

**Methods:**

A retrospective cohort study was conducted of preterm [<37 weeks gestational age (GA)] and/or LBW neonates (<2,500 g) born with a diagnosis of CHD, which requires invasive cardiac intervention (surgery or catheter) within their first year of life. Patients born between 2016 and 2020 and treated in three European pediatric heart centers were included.

**Results:**

A total of 308 neonates (51% male) with CHD were included. Of those, 237 (77%) were born preterm, 259 (84%) were LBW, and 188 (61%) were both. The median GA was 35.4 weeks (interquartile range 33.3–36.9) and the mean birth weight was 2,016 ± 580 g. CHD was categorized as simple (12%), moderate (64%), or severe (24%). The overall complication rate was 45% and was highest in patients with severe CHD (*p* = 0.002). One-year mortality (19%) was associated with severe CHD, low relative birth weight in patients with genetic diagnoses, and low GA at birth, whereas GA at birth significantly impacted survival only after 3 months of life.

**Conclusions:**

The high morbidity and mortality in preterm and LBW neonates with CHD reflect their complexity and consequent limited treatment feasibility.

## 1 Introduction

Congenital heart disease (CHD) is the most common congenital birth defect, affecting approximately 1% of live births (1). Patients with CHD are at increased risk for poor intrauterine growth (2) leading to low birth weight (LBW), as well as for spontaneous preterm birth when compared with the general population (3, 4). The causes may be multifactorial and include placental abnormalities, genetic comorbidities, and/or fetal distress (5).

Newborns experiencing both CHD and prematurity or LBW face an increased risk of medical complications. These patients constitute a complex patient population, further challenged by the underlying causes and effects of prematurity or LBW alone. While cardiac surgery has advanced significantly, there are still technical limitations to the procedures that can be performed in LBW neonates and, as such, watchful waiting may be needed, even under critical clinical conditions, until they have reached an adequate weight and maturity. Furthermore, genetic diagnoses are frequently associated with CHD (6) and may further increase the risk of medical complications.

There is a lack of contemporary data from Europe providing evidence for the increased medical burden in this population. Evaluating the current situation for this vulnerable group is crucial for devising future intervention strategies aimed at enhancing patient outcomes.

We thus aimed to describe the clinical course and 1-year mortality rates of a European patient cohort of preterm and/or LBW patients with CHD born between 2016 and 2020. We hypothesized that CHD severity has an impact on these outcome parameters and therefore stratified the results for simple, moderate, and severe CHD. Furthermore, risk factors for 1-year mortality were assessed.

## 2 Methods

### 2.1 Study design and patient population

We conducted a retrospective data analysis of preterm [<37 weeks of gestational age (GA)] and/or LBW neonates (<2,500 g) with a diagnosis of CHD, which requires invasive cardiac intervention(s) within the first year of life, either by cardiac surgery or by catheter intervention. Neonates born between 1 January 2016 and 31 December 2020 were included, irrespective of an underlying genetic diagnosis. Patients who died before an intended intervention were also included. Neonates with isolated ligation of patent ductus arteriosus were excluded. The data analysis was performed on behalf of the European Association Brain in Congenital Heart Disease (European ABC) within three pediatric heart centers (German Heart Center Munich, Germany; University Medical Center Utrecht, The Netherlands; and University Children's Hospital Zurich, Switzerland). The European ABC is a research consortium, funded by a grant of the European Society of Pediatric research of four pediatric heart centers focusing on the risk factors and determinants of neurodevelopmental outcome of children with complex types of CHD (7, 8).

### 2.2 Ethics

The institutional Medical Ethics Committees reviewed and approved this research (Munich: 334-21 S-EB, Medical Research Ethics Committee of the University Medical Center Utrecht/NedMec: No. 20/600, Kantonale Ethikkomission Zürich: BASEC No. 2021-00345). In Zurich, general parental informed consent was obtained for the further use of health-related data and biological samples for research and teaching. In Munich and Utrecht, consent was waived due to the use of anonymized data. Study data were collected in 2022 from electronic patient records and entered into an electronic data capturing tool, Castor™ (9).

### 2.3 Cardiac diagnosis

Diagnoses of CHD were categorized according to (1) Clancy score (10), (2) cyanotic or acyanotic type of CHD, and (3) simple, moderate, and severe types of CHD using a modified classification according to the European Society of Cardiology (ESC) guidelines for the grown-up CHD (GUCH) population (11). Minor modifications were necessary to adjust for the neonatal population. The modified classification (Supplementary Table SI) included simple CHD defined as isolated mild-to-moderate aortic, mitral, and pulmonary valve anomalies as well as isolated small-to-moderate left-to-right shunt CHD, such as atrial septal defect (ASD) or ventricular septal defect (VSD). Moderate CHD included severe aortic and pulmonary valve disease, large left-to-right shunt CHD, coarctation of the aortic arch, tetralogy of Fallot, simple transposition of the great arteries (TGA), and coronary artery anomalies. Severe CHD included common duct-dependent types in CHD, such as single-ventricle CHD requiring staged procedure until Fontan palliation, pulmonary atresia (±VSD), complex aortic arch anomalies from aortic arch hypoplasia to interrupted aortic arch, common arterial trunk, complex forms of TGA including double-outlet right ventricle (DORV)/TGA, other abnormal atrioventricular (AV) or ventriculoarterial (VA) connections, and neonatal Ebstein anomaly.

### 2.4 Clinical parameters

Demographic and clinical data were collected, including sex, gestational age and birth weight, birth mode and location (tertiary vs. non-tertiary care center), maternal age, antenatal corticosteroids, Apgar score at 5 min, and umbilical cord arterial pH. Furthermore, CHD and genetic diagnosis, timepoint of CHD diagnosis (pre- vs. postnatal), age and weight at first cardiac intervention, type of first cardiac intervention, complications, and mortality within the first year of life were of interest.

Weight was transformed into *z*-scores using GA and sex-specific data provided by Fenton and Kim (12) for birth data and by the National Center for Health Statistics (USA) (13) for neonatal and infant data. Small for GA (SGA) was defined as a birth weight *z*-score below −1.28. The first cardiac intervention was defined as the first cardiovascular surgery with or without cardiopulmonary bypass or the first catheter-based intervention, such as balloon dilatation of stenotic valves, if no cardiac surgery occurred within the first year. The risk of surgical procedure was classified according to the RACHS-1 score (14).

We created a dichotomous parameter “overall complication,” which was defined as at least one of the following diagnoses: (1) bronchopulmonary dysplasia (defined as additional oxygen at 36 weeks GA); (2) neurological morbidity (defined as one or more of the following, depicted in ultrasound or magnetic resonance imaging (MRI) data: intraventricular hemorrhage (IVH) Grade III–IV, cystic periventricular leukomalacia, posthemorrhagic ventricular dilatation with need for drainage, stroke or major intracranial non-IVH bleeding); (3) necrotizing enterocolitis (NEC) Bell stage II–III; (4) sepsis defined as C-reactive protein (CRP) >20 mg/L and positive blood culture or ≥5 days of antibiotics); (5) need for cardiopulmonary reanimation (CPR); or (6) use of extracorporeal membrane oxygenation (ECMO).

Mortality was classified as neonatal (<28 days of life), pre/intra- or post-intervention (within the first year of life). The cause of death was categorized into cardiac (e.g., cardiovascular failure), non-cardiac (e.g., sepsis, genetic disease), or combined (e.g., treatment withdrawal due to complicated post-intervention course).

### 2.5 Statistics

Continuous data were presented as mean and standard deviation (SD) for normally distributed data and as median and interquartile range (IQR) for non-normally distributed data. Categorical data were presented as number and percentage. The normality of variables was assessed by visual inspection of distribution on Q-Q plots and histograms. Missing data of the reported variables were <5% unless otherwise specified. Differences within CHD categories were assessed using ANOVA (parametric data), Kruskal–Wallis test (non-parametric data), and chi-square test (categorical data).

Survival probability was visualized using a Kaplan–Meier curve. Cox regression was applied to evaluate differences in survival between severity grades of CHD. In addition, adjusted Cox regression was conducted, including the variables CHD severity, genetic diagnosis, GA at birth, *z*-score of birth weight, and interaction of genetic diagnosis * *z*-score of birth weight. Variables were chosen by clinical implication; interaction was added after significance testing. Proportional hazard assumption was tested using Schoenfeld residuals. As GA at birth did not meet the assumption, a model using time-varying coefficients was applied.

The alpha significance level was set at 0.05 for this exploratory analysis. Statistical analyses were performed using R (version 4.1.2, The RSoftware Foundation for Statistical Computing, Vienna, Austria).

## 3 Results

A total of 308 patients with CHD were enrolled at three European pediatric heart centers. The median GA was 35.4 weeks (IQR 33.3–36.9) and mean birth weight was 2,016 ± 580 g, with a corresponding mean *z*-score of 0.94 ± 1.15. Of the 308 patients, 237 (77%) were born preterm, 259 (84%) were LBW, and 188 (61%) were both. A total of 127 (41%) patients were SGA.

### 3.1 Baseline characteristics

Baseline clinical, obstetric, and demographic parameters were tested for differences according to CHD severity. Details are shown in Table 1. CHD severity was simple in 37 (12%) patients, moderate in 196 (64%) patients, and severe in 75 (24%) patients. The most frequent diagnoses in simple CHD were isolated left-to-right shunts (*n* = 19) and isolated pulmonary valve defects (*n* = 11); in moderate CHD, more complex left-to-right shunts (*n* = 84) and coarctation of the aorta or hypoplastic aortic arch (*n* = 39) were seen; and in severe CHD, pulmonary atresia (*n* = 29) and single-ventricle or other complex anomalies of AV or VA connections (*n* = 26) were observed. Details of diagnoses and corresponding treatment are provided in the supplement (Supplementary Table SII).

**Table 1:** Baseline characteristics according to CHD severity.

CHD category	Simple	Moderate	Severe	*p*-value
*n*	37	196	75	
Sex, male (%)	14 (38)	98 (50)	45 (60)	0.079
GA at birth, weeks [median (IQR)]	35.0 (31.4–36.6)	35.3 (33.0–36.6)	36.6 (34.4–7.5)	**0**.**001**
Preterm	30 (81)	161 (82)	46 (61)	**0**.**001**
* *<28.0 (%)	2 (5)	9 (5)	0	
28.0 to <32.0 (%)	9 (24)	27 (14)	6 (8)	
32.0 to <34.0 (%)	7 (19)	21 (11)	7 (9)	
34.0 to <37.0 (%)	12 (32)	104 (53)	33 (44)	
≥37.0 (%)	7 (19)	35 (18)	29 (39)	** **
Birth weight, g [mean (SD)]	1,964 (538)	1,965 (603)	2,174 (510)	**0**.**024**
Birth weight, *z*-score [mean (SD)]	−0.52 (1.39)	−0.95 (1.07)	−1.10 (1.20)	**0**.**042**
LBW (<2,500 g) (%)	31 (84)	167 (85)	61 (81)	0.737
<1,000 g (%)	2 (5)	16 (8)	1 (1)	
1,000–<1,500 g (%)	5 (14)	28 (14)	7 (9)	
1,500–<2,500 g (%)	24 (65)	123 (63)	53 (71)	
≥2,500* *g (%)	6 (16)	29 (1)	14 (19)	
SGA (%)	9 (24)	80 (41)	38 (51)	**0**.**028**
Preterm and SGA (%)	2 (5)	50 (26)	11 (15)	**0**.**008**
Preterm and LBW (%)	24 (65)	132 (67)	32 (43)	**0**.**001**
Birth mode (%)				0.314^a^
Spontaneous delivery (%)	5 (14)	48 (25)	18 (24)	** **
Cesarean section (%)	24 (65)	128 (65)	52 (69)	** **
Induced labor (%)	2 (5)	4 (2)	5 (7)	** **
Unknown (%)	6 (16)	16 (8)	0	** **
Birth in tertiary clinic (%)	22 (60)	133 (68)	56 (75)	0.259
Maternal age [mean (SD)]	33.7 (4.5)	33.0 (5.5)	30.8 (5.0)	**0**.**005**
Antenatal corticosteroids^b^ (%)	14 (38)	47 (24)	15 (20)	0.063
Apgar score at 5 min^c^ [median (IQR)]	8 (7–8)	8 (7–9)	8 (7–9)	0.555
arterial pH, umbilical cord [mean (SD)]	7.30 (0.07)	7.29 (0.09)	7.28 (0.08)	0.532
Diagnosis				
Clancy Class				**<0**.**001**
1	35 (95)	140 (71)	35 (47)	
2	2 (5)	55 (28)	9 (12)	
3	0	1 (1)	13 (17)	
4	0	0	18 (24)	
Cyanotic CHD	1 (3)	52 (27)	66 (88)	**<0**.**001**
Duct-dependent circulation	5 (14)	72 (37)	58 (77)	**<0**.**001**
Prenatal diagnosis	13 (37)	87 (46)	53 (71)	**<0**.**001**
Genetic anomaly	11 (31)	66 (34)	21 (29)	0.729

min, minute.

Significant values are given in bold (*p* < 0.05).

^a^ Difference in birth mode was tested after removing unknown cases.

^b^ Antenatal corticosteroid information was not available in 26 patients.

^c^ Apgar score was not available in 31 patients.

Patients with severe CHD were older at birth and more frequently born at term compared to patients with simple and moderate CHD. No very-preterm patient (GA <28 weeks) with severe CHD was part of our study population. Patients with severe CHD had the highest absolute birth weight, but the lowest relative birth weight (*z*-score) and highest frequency of SGA. The percentage of patients with LBW was comparable between the three CHD severity groups, in the range of 81%–85%. Mothers were youngest in patients with severe CHD. Patients with severe CHD were most frequently diagnosed prenatally.

The severity of CHD was associated with the Clancy classification, cyanosis, and duct-dependency. The rate of genetic diagnosis was comparable between the three CHD severity groups, in the range of 29%–34%. Trisomy 21 (*n* = 36, 12%) was the most frequent genetic anomaly. Details on genetic diagnoses are available in Supplementary Table SIII.

### 3.2 First cardiac intervention

Of the 286 (93%) patients surviving until the first intervention, 90% underwent cardiac surgery (*n* = 256), whereas 10% had catheter-based interventions only within their first year of life (*n* = 31). Patients with severe CHD were treated earliest (concerning both corrected age [postmenstrual age (PMA) and uncorrected (chronological) age] at the lowest absolute body weight and underwent the highest-risk surgeries according to the RACHS-1 score (for all, *p* < 0.001). The mean relative body weight (*z*-score) at first intervention was more than 2 standard deviations below the normative value for all CHD severity grades and showed no evidence for a significant difference between the groups (Table 2).

**Table 2:** Details on first cardiac intervention according to CHD severity.

CHD category	Simple	Moderate	Severe	*p-*value
*N*	37	185	64	
Chronological age at intervention, days [median (IQR)]	119 (42–218)	89 (20–157)	11 (6–60)	**<0**.**001**
PMA at intervention, weeks [median (IQR)]	54.4 (38.0–66.6)	47.0 (38.6–57.2)	38.6 (37.0–41.8)	**<0**.**001**
Body weight at intervention, g [median (IQR)]	4,460 (2,600–6,080)	3,840 (2,400–5,270)	2,500 (2,185–3,070)	**<0**.**001**
<3,000 g	12 (32)	70 (39)	45 (70)	**<0**.**001**
Body weight, *z*-score [mean (SD)]	−2.03 (1.46)	−2.13 (1.43)	−2.26 (1.14)	0.685
Type of intervention: surgery [*n* (%)]	23 (62)	174 (94)	59 (92)	**<0**.**001**
Surgical risk				**<0**.**001**
* *RACHS 1	6 (16)	13 (7)	0	
RACHS 2	12 (32)	80 (44)	6 (10)	
RACHS 3	4 (11)	77 (42)	32 (51)	
RACHS 4	1 (3)	3 (2)	12 (19)	
RACHS 5	0	0	2 (3)	
RACHS 6	0	0	6 (10)	
RACHS NA	0	1 (1)	1 (2)	
Definite repair [*n* (%)]	33 (89)	141 (76)	10 (16)	**<0**.**001**

PMA, postmenstrual age; RACHS, risk-adjustment for congenital heart surgery (14).

Significant values are given in bold (*p* < 0.05).

### 3.3 Complications

The overall complication rate of the whole cohort was 45% and was associated with CHD severity (Table 3). In patients with severe CHD, cardiac complications (CPR or ECMO) as well as post-intervention sepsis were most frequent. In contrast, the rates of pre- and post-intervention neurological complications and NEC showed no evidence of a significant difference within the CHD severity groups; however, they were rare or absent in patients with simple CHD.

**Table 3:** Complications and mortality according to CHD severity.

CHD category	Simple	Moderate	Severe	*p*-value
*N*	37	196	75	
Overall complication [*n* (%)]	11 (30)	82 (42)	46 (61)	**0**.**002**
Pre-interventional complications				
* *Bronchopulmonary dysplasia	4 (11)	26 (13)	7 (9)	0.653
Neurological complication [*n* (%)]	0	16 (8)	4 (5)	0.162
NEC, Bell stage II or III	2 (5)	12 (6)	10 (13)	0.119
Sepsis	5 (14)	31 (16)	12 (16)	0.933
CPR	1 (3)	16 (8)	12 (16)	**0**.**047**
Post-interventional complications				
*N*	37	185	64	
Neurological complication [*n* (%)]	0	8 (4)	7 (9)	0.068
NEC, Bell stage II or III	0	8 (4)	7 (9)	0.068
Sepsis	5 (14)	24 (12)	23 (31)	**0**.**001**
CPR	4 (11)	13 (7)	16 (21)	**0**.**002**
ECMO	1 (3)	5 (3)	8 (11)	**0**.**014**
Overall mortality [*n* (%)]	2 (5)	25 (13)	31 (41)	**<0**.**001**
Neonatal mortality (<28 days) [*n* (%)]	1 (50)	7 (29)	17 (55)	0.161
Pre-interventional mortality [*n* (%)]	0	11 (6)	11 (15)	**0**.**007**
Chronological age at death, days [median (IQR)]	—	41 (13–90)	8 (2–11)	**0**.**006**
PMA at death, weeks [median (IQR)]	—	41.0 (35.1–43.6)	38.0 (36.9–39.6)	0.844
Cause of death [*n* (%)]	—			—
Cardiac	—	3 (27)	4 (36)	
Combined	—	1 (9)	3 (27)	
Non-cardiac	—	6 (55)	4 (36)	
Unknown	—	1 (9)	—	
Decision for comfort care [*n* (%)]	—	4 (36)	8 (73)	–
Death due to delayed intervention	—	2 (18)	—	—
Survived until intervention, *n*	37	185	64	
Deceased during intervention [*n* (%)]	—	—	—	
Post-interventional Mortality [*n* (%)]	2 (5)	14 (8)	20 (31)	**<0**.**001**
Chronological age at death, days [median (IQR)]	34 (21–46)	55 (37–154)	92 (25–135)	0.441
PMA at death, weeks [median (IQR)]	35.9 (35.7–36.0)	43.4 (41.7–54.3)	47.9 (39.3–52.2)	0.088
Cause of death [*n* (%)]				0.862
* *Cardiac	1 (50)	5 (36)	9 (45)	
Combined	1 (50)	6 (43)	9 (45)	
Non-cardiac	0	3 (21)	2 (10)	
Decision for comfort care [*n* (%)]	0	3 (21)	7 (37)	0.409
Treatment complication [*n* (%)]	1 (50)	3 (21)	5 (26)	0.685
Mortality within 30 days *post-*intervention [*n* (%)]	2 (100)	7 (54)	9 (45)	0.325

PMA, postmenstrual age.

Significant values are given in bold (*p* < 0.05).

Pre-intervention cranial ultrasound data for the assessment of neurological complications (as defined in methods) were available in 76% of patients and cerebral MRI in 12% of patients, whereas post-intervention cranial ultrasound was conducted in 47% of patients and cerebral MRI in 13% of patients.

### 3.4 Mortality

The overall mortality rate in the first year of life was 19% (*n* = 58). A total of 22 patients died before the intervention, most frequently due to non-cardiac causes (45%), and 36 patients died after the intervention, most frequently due to cardiac (42%) or combined causes (cardiac and non-cardiac, 44%). The decision for comfort care was made in 12 (55%) pre-intervention and 10 (28%) post-intervention cases. Of the post-intervention deaths, 50% occurred within 30 days after the first cardiac intervention, and 25% of post-intervention deaths were treatment-related. Total, pre-, and post-intervention mortality rates differed across the CHD severity groups and were highest in patients with severe CHD (see Table 3).

**Figure 1:**
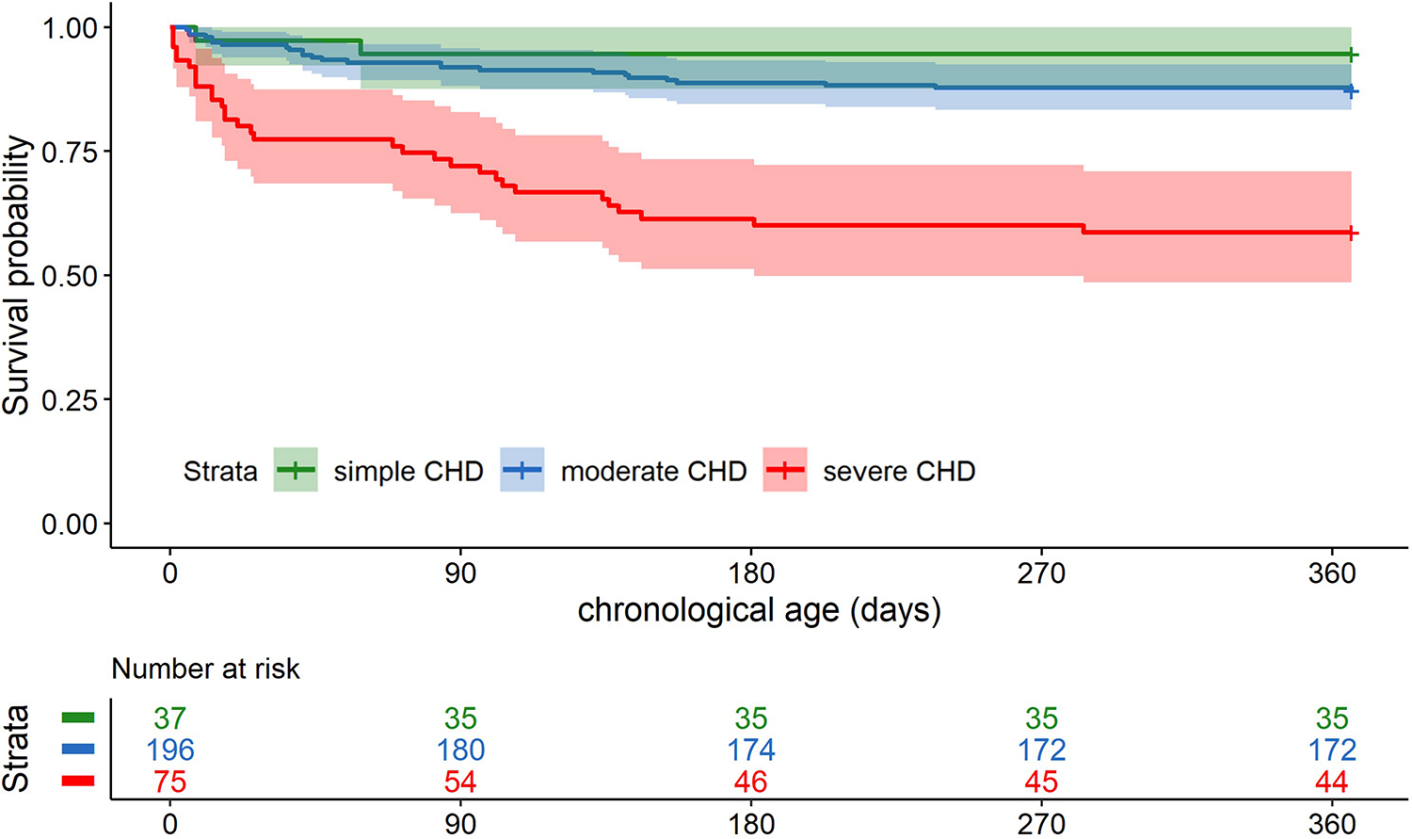
Kaplan–Meier curve for 1-year survival according to CHD severity.

The 1-year survival rate of the total cohort (*n* = 307, one patient was excluded due to missing timepoint of death) was 82% [95% confidence interval (CI) 77–86]. For simple CHD, the survival rate was 95% (95% CI: 88–100); for moderate CHD, the survival rate was 88% (95% CI: 83–92); for severe CHD, the survival rate was 59% (95% CI: 49–71). The 1-year survival curve for the CHD severity groups is shown in Figure 1. The survival probability of patients with simple and moderate CHD was significantly higher than that of patients with severe CHD [estimated hazard ratio (HR) simple vs. severe 0.10 (95% CI: 0.02–0.44), *p* = 0.002; moderate vs. severe 0.25 (95% CI: 0.15–0.43), *p* < 0.001]. No evidence for a difference in survival probability was found for simple versus moderate CHD.

In addition, the effects of genetic diagnosis, GA at birth, and *z*-score of birth weight on 1-year survival were evaluated in an adjusted Cox regression. CHD severity and genetic diagnosis had significant time-independent associations with 1-year survival (for details, see Table 4). Diagnoses of simple or moderate CHD (compared to severe CHD) were still associated with increased 1-year survival. Moreover, in the presence of a genetic diagnosis, a higher relative birth weight was associated with increased 1-year survival. The effect of GA at birth on 1-year survival varied with the chronological age of the patients. While no evidence for significant association with survival was found in the first 90 days of life, a higher GA at birth was significantly associated with increased survival after 90 days of life.

**Table 4:** Adjusted cox regression with time-varying coefficients for gestational age.

Parameter	Hazard ratio	95% CI	*p*-value
Simple CHD type (vs. severe)	0.10	0.02–0.43	**0**.**002**
Moderate CHD type (vs. severe)	0.24	0.14–0.42	**<0**.**001**
Presence of genetic anomaly	1.32	0.63–2.77	0.469
Birth weight, *z*-score	1.14	0.81–1.61	0.443
Genetic anomaly* ** birth weight, *z*-score (interaction term)	0.60	0.37–0.95	**0**.**030**
GA at birth (weeks), time-period 1–14 days of life	0.99	0.83–1.18	0.903
GA at birth (weeks), time-period 15–90 days of life	0.98	0.84–1.15	0.821
GA at birth (weeks), time-period 91–365 days of life	0.80	0.71–0.91	**<0**.**001**

Significant values are given in bold (*p* < 0.05).

## 4 Discussion

In our study, we described the clinical course and 1-year mortality in patients with CHD complicated by preterm birth or LBW at three European pediatric heart centers. The lower 1-year survival of our cohort was associated with a diagnosis of severe CHD, low relative birth weight in patients with genetic diagnosis, and low GA at birth.

Our cohort of preterm and/or LBW patients with CHD was at high risk for 1-year mortality, with a rate of 19%. A similar rate was reported by Laas et al. (15), with a 1-year mortality of 17.9% in preterm CHD, which was 3.8-fold higher than in term born patients with CHD.

The 1-year mortality of the severe CHD group was the highest with 41%. Comparable data are rare. One study reported a peri-intervention mortality of 38% in patients with hypoplastic left heart syndrome born at LBW (born between 2000 and 2004) (16). Both rates correspond approximately to the reported 5-year mortality in term born, average-weighted patients with isolated severe CHD (42%) (17).

As described by others, the severity of CHD is an important factor affecting mortality in both term and preterm patients (17, 18). We found severe CHD to be associated with worse outcomes, but no evidence for differences between simple and moderate CHD. The effect of CHD severity was strong, showing that patients with simple CHD have a 90% reduced risk of death, while those with moderate CHD have a 76% reduced risk of death compared with severe CHD.

The impact of GA at birth on survival varied over the first year of life, meaning that an association between lower GA and impaired survival was only found in patients surviving to 3 months of age. In fact, in this group of patients, an increase of 1 week in GA at birth reduced the risk of death by 20%. Other factors, such as CHD severity and relative birth weight, in patients with genetic diagnosis seem to play a more important role in the early postnatal period. Prematurity is associated with chronic diseases (e.g., bronchopulmonary dysplasia), which may have led to this accentuated mid- and long-term mortality.

The survival of patients with isolated CHD was independent of relative birth weight. On the other hand, patients with a genetic diagnosis and low relative birth weight were at increased risk for mortality in the first year of life.

A survival analysis using a multivariable model was also assessed by Best et al. (17), evaluating a population-based register including both preterm and term born patients with CHD in a specific geographic area born between 1985 and 2003. They found CHD severity, low GA at birth, low relative birth weight, extracardiac anomalies, and earlier year of birth to be independently associated with 5-year mortality.

A single-center study of patients born between 2015 and 2017 (18) found severity of CHD, LBW, and 1 min Apgar score to be independent risk factors for in-hospital mortality in a cohort of both preterm and term infants with CHD, whereas GA at birth did not affect survival.

In comparison to our study, these two studies analyzed a cohort of both preterm and term born patients with CHD, and furthermore used categorical variables for GA at birth/prematurity (very preterm vs. moderately preterm vs. term vs. post-term/preterm vs. non-preterm) and birth weight (*z*-score <−1 vs. −1 to ≤1 vs. >1/LBW vs. non-LBW). Compared to that, the strength of our study was that it assessed the risk factors in a more detailed way, using continuous variables where appropriate. This may have led to the deviations in our results, e.g., that GA at birth is only predictive after 3 months of life, and the *z*-score of birth weight is predictive only in patients with a genetic diagnosis.

Though our study, as well as the two studies mentioned above, evaluated risk factors known at birth to enable proper survival modeling, other studies investigated the association of variables of the clinical course with mortality. Several studies found ventilation days, risk score of cardiac surgery, weight at surgery, or prevalence of complications (CPR, use of ECMO) to be associated with mortality (16, 19–21) in cohorts of vulnerable LBW or premature patients with CHD.

As a strength of the present study, we investigated this high-risk cohort, including patients with a genetic diagnosis. The rate of genetic comorbidity was similar in the CHD severity groups. With a prevalence of approximately 30%, it was higher than reported for overall CHD cohorts (irrespective of GA at birth), which is approximately 11%–17% (6, 22). A prevalence of genetic diagnoses in preterm patients with CHD approximately twice as likely as in term born patients with CHD was already described in another study, but with lower absolute percentages (13.2% in preterm infants vs. 5.1% in full-term infants) (15), which may be impacted by the genetic testing frequency of the included birth years (2005–2008). Currently, genetic anomalies are diagnosed more frequently because of increased screening and improvements in diagnostic precision (22). However, our prevalence of genetic diagnoses might be biased by the number of tests performed, which was not analyzed.

We described patient characteristics and clinical course according to CHD severity, which is one of the most important influencing factors for survival in patients with CHD (18). We found several strong associations of CHD severity and clinical course in the patients, verifying the evidence of our CHD severity categorization, which adheres to an already-published categorization used in the GUCH cohort (11).

We found that relative birth weight is lowest in patients with severe CHD, reflecting the impact of cardiac disease on body growth. Even though absolute birth weight was highest in patients with severe CHD, this group underwent their first cardiac intervention at the lowest absolute weight, due to the early time point of the intervention (lowest chronological and gestational age at intervention). The highest absolute birth weight in patients with severe CHD of our cohort may be related to their highest GA at birth. As patients with severe CHD were most frequently SGA, they met the study inclusion criteria of LBW not being preterm more often than simple/moderate CHD. Nevertheless, we report a low absolute number of very preterm patients with severe CHD, none born before 28 weeks, which may be caused by an increased frequency of comfort care treatment without transferal to a specialized hospital.

In our cohort, patients with simple CHD were born to the oldest mothers and, on the contrary, patients with severe CHD to the youngest mothers. The first finding is in line with other publications reporting an association of advanced maternal age with simple heart defects (23, 24), where chromosomal anomalies are determined to be an underlying factor (23, 25, 26). Interestingly, young maternal age was associated with severe CHD. This was also found by Mamasoula et al. (maternal age ≤24 years associated with severe CHD) (23), who discussed the possibility of socioeconomic factors, including poor diet, smoking, and alcohol or drug use as the etiology of severe CHD in this population. Nevertheless, the etiology of the association of severe CHD and young mothers with a mean age of 31 ± 5 years in our cohort remains unknown.

Pre- and post-intervention cardiac complications (need for CPR, use of ECMO) were associated with CHD severity. Interestingly, the frequency of non-cardiac pre-intervention complications was very similar across the CHD groups; this may be influenced by the lower GA and/or longer pre-intervention period in patients with less severe CHD. Post-intervention non-cardiac complications differed more strongly within CHD severity types, reflecting the complexity of the intervention and its impact on the post-intervention clinical course.

The present study has some limitations. In pre-intervention deaths, in particular, the decision for comfort care was frequent (55%). Decisions may have been based on genetic diagnoses (e.g., trisomy 18) or the occurrence of devastating complications. Nevertheless, parents have the option to decide for comfort care in the absence of these reasons, adding a bias to our survival analysis if therapeutic options were declined. Furthermore, the abortion rate was not evaluated and patients in a palliative setting born at a non-tertiary care center were not included in this study.

An attempt was made to overcome heterogeneity issues by using a multicenter approach and CHD severity categorization. However, limitations remain because of the variety of influencing factors (e.g., no differentiation between genetic diagnoses of known vs. unknown clinical significance). To account for this diversity, and achieve better generalizability, a comparison with term born patients with a birth weight ≥2.5 kg for direct calculation of additive risk by prematurity and/or LBW would be beneficial. Furthermore, the causes of prematurity were not reported and may be linked to either the mother, child, or both.

The applied diagnostic approach of NEC with Bell staging has limitations in accuracy resulting in a risk of overdiagnosis and overtreatment (27). Moreover, our definition of sepsis did not include organ failure due to the retrospective design of the study with the lack of data availability. As a consequence, the incidence of both complications may be overestimated.

Pulmonary hypertension was not reported as a complication due to the lack of retrospective differentiation between cardiac and pulmonary etiology, but bronchopulmonary dysplasia was included in the analysis.

The alpha significance level was set at 0.05 and not adjusted for multiple testing because of the exploratory design of the study.

In summary, the clinical course of patients with both CHD and prematurity or LBW is characterized by the interplay of multiple pathologies, with intervention limitations imposed by physiological vulnerabilities. In this study, severe CHD, low relative birth weight in patients with a genetic diagnosis, and low GA at birth were associated with increased 1-year mortality. However, the influencing factors are numerous and therapeutic decisions need to be tailored to the individual patient.

## Data Availability

The original contributions presented in the study are included in the article/**Supplementary Materials**, further inquiries can be directed to the corresponding author.

## Group members of the European Association Brain in Congenital Heart Disease

Mirielle N. Bekker: Division of Woman and Baby, University Medical Center Utrecht, Utrecht University, Utrecht, Netherlands. Manon J. N. L. Benders, Floris Groenendaal, Corine Koopman-Esseboom, Maaike Nijman, and Raymond Stegeman: Department of Neonatology, Wilhelmina Children's Hospital, Utrecht University, Netherlands. Alexandra Bonthrone, Andrew Chew, Serena J. Counsell, Daniel Cromb, Barat Gal-Er, Christopher J. Kelly, and Kuberan Pushparajah: Centre for the Developing Brain, School of Biomedical Engineering and Imaging Sciences, King's College London, UK. Rian Bosch, Sergei Chin On, Nicolaas J. G. Jansen, and Valerie D. Slooff: Department of Pediatric Intensive Care, University Medical Center Utrecht, Utrecht, Netherlands. Johannes M. P. J. Breur, Nathalie H. P. Claessens, Maaike Nijman, and Raymond Stegeman: Department of Pediatric Cardiology, Wilhelmina Children's Hospital, Utrecht University, Netherlands. Hitendu Dave, Maria Feldmann, Raimund Kottke, Beatrice Latal, and Céline Steger: Children's Research Center, University Children's Hospital Zurich, Zurich, Switzerland. Hitendu Dave: Division of Congenital Cardiovascular Surgery, Pediatric Heart Center, Department of Surgery, University Children's Hospital Zurich, University of Zurich, Switzerland. Maria Feldmann and Beatrice Latal: Child Development Center, University Children's Hospital Zurich, University of Zurich, Switzerland. Monique C. Haak: Department of Obstetrics and Fetal Medicine, Leiden University Medical Center, Leiden, Netherlands. Nicolaas J. G. Jansen and Raymond Stegeman: Department of Pediatrics, Beatrix Children's Hospital, University Medical Center Groningen, Netherlands. Raimund Kottke: Department of Diagnostic Imaging, University Children's Hospital Zurich, University of Zurich, Switzerland. Céline Steger: Pediatric Cardiology, Pediatric Heart Center, University Children's Hospital, University of Zurich, Zurich, Switzerland.
