# Supplementary material for: Morbidity and mortality in premature or low birth weight patients with congenital heart disease in three European pediatric heart centers between 2016 and 2020
Source: Front Pediatr. 2024 Apr 11;12:1323430. doi: 10.3389/fped.2024.1323430 (PMC11043489; doi:10.3389/fped.2024.1323430)
Supplement: Supplementary file 1 [file Datasheet1.docx]

Supplementary Material

# Supplementary Tables

Table I. Modified classification of congenital heart disease (adapted from Baumgartner et al.(11))

|  | **Simple CHD** | **Moderate CHD** | **Severe CHD** |
| --- | --- | --- | --- |
| Aortic valve | Isolated mild-to-moderate aortic valve disease / bicuspid aortic valve disease | Moderate-to-severe aortic stenosis (subvalvular, supravalvular) | Critical (duct-dependent) aortic valve stenosis |
| Pulmonary valve | Isolated mild-to-moderate pulmonary valve disease | Moderate-to-severe pulmonary stenosis (infundibular, valvular, supravalvular) and peripheral pulmonary stenosis | Critical (duct-dependent) pulmonary valve stenosis |
| Mital valve | Isolated mild-to-moderate mitral valve disease (except parchute valve, cleft leaflet) | Moderate-to-severe mitral valve stenosis and/or regurgitation | Mitral atresia (duct-dependent) |
| Left-to-right shunt | Isolated small-to-moderate left-to-right shunt CHD, i.e. ASD, or VSD, or PDA, or other (just one entity) | Large left-to-right shunt CHD, i.e. ASD, and/or AVSD, and/or VSD, and/or PDA, and/or sinus venosus defect +/- PAPVD (more than one entity), AP window |  |
| Pulmonary vein connection | Isolated pulmonary venous connection (PAPVD without ASD) | PAPVD with ASD;  total anomalous pulmonary venous connection (TAPVD) without restriction | Total anomalous pulmonary venous connection (TAPVD) with restriction |
| Aortic arch | Double aortic arch | CoA and hypoplastic aortic arch | Critical CoA (duct-dependent) Interrupted aortic arch |
| Right ventricular outflow tract | Double chambered right ventricle with VSD | Tetralogy of Fallot (including DORV, Fallot type),  DORV, VSD type | Pulmonary atresia  (all forms: PA+IVS, PA/VSD, complex PA/VSD/MAPCA’s))  DORV, complex (TGA type and other) |
| TGA |  | TGA, simple | TGA, complex |
| Truncus arteriosus communis |  |  | Truncus arteriosus communis |
| Coronary artery |  | Coronary artery anomalies (ALCAPA, other) *without* severe heart failure | Coronary artery anomalies (ALCAPA, other) *with* severe heart failure |
| Tricuspid valve | Mild tricuspid dysplasia (non Ebstein) | Ebstein anomaly | Neonatal (severe) Ebstein anomaly (duct-dependent) |
| Other |  | Marfan / Turner syndrome (other HTAD), cardiomyopathies | Single Ventricle CHD (duct dependent) including DILV, Tricuspid/mitral atresia, HLHS, dysbalanced AVSD, others  Other complex abnormalities of AV and VA abnormal connections (heterotaxy syndrome, crisscross heart, other) |

ALCAPA: anomalous left coronary artery origin from pulmonary artery, AP: aortopulmonary, ASD: atrial septal defect, AV: atrio-ventricular, AVSD: atrioventricular septal defect, CHD: congenital heart disease, CoA: coarctation of the aorta, DILV: double inlet left ventricle, DORV: double outlet right ventricle, HLHS: hypoplastic left heart syndrome, HTAD: heritable thoracic aortic disease, IVS: intact ventricular septum, MAPCA: major aortopulmonary collateral artery, PA: pulmonary artery, PAPVD: partial anomalous pulmonary venous drainage, PDA: patent ductus arteriosus, TAPVD: total anomalous pulmonary venous drainage, TGA: Transposition of the Great Arteries, VA: ventriculo-arterial, VSD: ventricular septal defect

Table II. Detailed CHD diagnoses and primary invasive procedure.

|  | **Total,**  **n=** | **Death before any procedure, n=** | **Catheter procdure,**  **n=** | **Surgical procedure,**  **n=** |
| --- | --- | --- | --- | --- |
| **Simple CHD** |  |  |  |  |
| Aortic valve stenosis, moderate | 3 | - | 2 | 1 |
| Pulmonary valve disease, moderate | 11 | - | 10 | 1 |
| Left-to-right shunt CHD, small-moderate | 19 | - | 2 | 17 |
| Double aortic arch | 4 | - | - | 4 |
| **Moderate CHD** |  |  |  |  |
| Aortic valve stenosis, severe  (including subvalvular, supravalvular) | 3 | 2 | 1 | - |
| Pulmonary valve stenosis, severe (including infundibular, supravalvular), peripheral pulmonary artery stenosis | 13 | 2 | 5 | 6 |
| Left-to-right shunt CHD, large  (including AP window) | 84 | 5 | 2 | 77 |
| PAPVD with ASD  TAPVD without restriction | 5 | - | - | 5 |
| CoA and hypoplastic aortic arch | 39 | - | 1 | 38 |
| Tetralogy of Fallot  (including DORV, Fallot type),  DORV, VSD type | 38 | 1 | 1 | 36 |
| Simple TGA | 10 | - | 1 | 9 |
| Coronary artery anomalies (ALCAPA, other) without severe heart failure | 1 | - | - | 1 |
| Ebstein anomaly | 2 | 1 | - | 1 |
| Marfan / Turner syndrome (HTAD), cardiomyopathies | 1 | - | - | 1 |
| **Severe CHD** |  |  |  |  |
| Aortic valve stenosis, critical | 1 | - | 1 | - |
| Pulmonary valve stenosis, critical | 3 | - | 1 | 2 |
| CoA, critical Interrupted aortic arch | 2 | - | - | 2 |
| Pulmonary atresia  Complex DORV (TGA type and other) | 29 | 4 | 2 | 23 |
| Complex TGA | 4 | - | - | 4 |
| Truncus arteriosus communis | 6 | 1 | - | 5 |
| Ebstein anomaly, neonatal (severe) | 4 | 1 | - | 3 |
| Single Ventricle CHD,  other complex abnormalities of AV and VA abnormal connections | 26 | 5 | 1 | 20 |

ALCAPA: anomalous left coronary artery origin from pulmonary artery, AP: aortopulmonary, ASD: atrial septal defect, AV: atrio-ventricular, CHD: congenital heart disease, CoA: coarctation of the aorta, DORV: double outlet right ventricle, HTAD: heritable thoracic aortic disease, PAPVD: partial anomalous pulmonary venous drainage, TAPVD: total anomalous pulmonary venous drainage, TGA: Transposition of the Great Arteries, VA: ventriculo-arterial, VSD: ventricular septal defect

Table III. Genetic diagnoses according to severity of congenital heart disease.

| CHD category | simple | moderate | severe |
| --- | --- | --- | --- |
| Trisomy 21 (n=36) | 6 | 30 |  |
| Noonan syndrome (n=6) |  | 6 |  |
| Microdeletion 22q11 (n=4) |  | 1 | 3 |
| Williams-Beuren syndrome (n=3) |  | 3 |  |
| Alagille syndrome (n=2) |  | 1 | 1 |
| CHARGE syndrome (n=2) |  | 2 |  |
| Jacobsen syndrome (n=2) | 1 | 1 |  |
| Kabuki syndrome (n=2) |  |  | 2 |
| Trisomy 13 (n=2) |  | 1 | 1 |
| Trisomy 18 (n=2) |  | 2 |  |
| 17q12 duplication syndrome (n=1) |  | 1 |  |
| 46,XY/47,XYY mosaic with 15q11.2 deletion (n=1) |  | 1 |  |
| Atypical deletion 22q11.21 (n=1) |  | 1 |  |
| Cat-eye syndrome (n=1) |  |  | 1 |
| Chromosome anomaly 47, XYY (mosaic), suspected Ritscher Schinzel syndrome (n=1) |  | 1 |  |
| Consanguinity of parents (high suspicion for genetic anomaly with kidney agenesis and hypospadia, not tested) |  | 1 |  |
| Dandy Walker Malformation, trisomy 21 (n=1) |  | 1 |  |
| De novo mutation intron 5 gene CASZ1, 2 compounds heterozygosity gene CASKIN1, microdeletion of a sequence from chromosome 9 (n=1) |  |  | 1 |
| De novo mutation TCF 20 (n=1) |  |  | 1 |
| Deletion Chromosome 11q24.1q25 (region associated with Jacobson-Syndrome) (n=1) |  |  | 1 |
| Deletion syndrome 22q11.2 (n=1) |  | 1 |  |
| Fetal partial monosomy Xp (about 49 Mb deletion of chromosomal material of the bands Xpter until p11.23) (n=1) |  | 1 |  |
| Heterozygous mutation BRCA2 gene (translocation 15-22)(q22-q13) (n=1) |  | 1 |  |
| MEIS-2 mutation (n=1) |  |  | 1 |
| Microdeletion-syndrome 3p25.3 (heterozygous deletion of chromosomal material of about 82Kb) (n=1) |  | 1 |  |
| Mosaic Turner syndrome (n=1) |  | 1 |  |
| Mosaicism trisomy 9 (n=1) | 1 |  |  |
| Myofibrillar myopathy type 5 (n=1) |  | 1 |  |
| NOTCH 1 mutation (n=1) |  |  | 1 |
| Partial deletion of chromosome 11 (11q14.3q22.3) (n=1) | 1 |  |  |
| Partial mosaic trisomy 16 (n=1) | 1 |  |  |
| Partial trisomy 21 (n=1) |  |  | 1 |
| Partial trisomy 5p, partial monosomy 21q (n=1) |  |  | 1 |
| Partial trisomy 6 (n=1) |  |  | 1 |
| Partial trisomy 6q (n=1) | 1 |  |  |
| Pathogenic mutation GDF1 (n=1) |  | 1 |  |
| Prader Willi syndrome (n=1) |  |  | 1 |
| Robertsonsche trisomy 21 (n=1) |  | 1 |  |
| SCN5A-mutation, Brugada syndrome (n=1) |  |  | 1 |
| Sequence variant MYH6 gene (n=1) |  |  | 1 |
| Suspected CHARGE syndrome, pericentric inversion chromosome 1 (n=1) |  | 1 |  |
| Suspected Frank ter Haar syndrome (FTHS) (n=1) |  | 1 |  |
| Suspected Koolen de Vries syndrome (n=1) |  | 1 |  |
| Tricho-hepato-enteric syndrome (mutation in TCC 37) (n=1) |  | 1 |  |
| Turner syndrome (monsomy 45 X) (n=1) |  | 1 |  |
| Variant of unknown significance: exon 24 of RANBP2-gene (n=1) |  |  | 1 |
| Wolf-Hirschorn syndrome (n=1) |  |  | 1 |
